# Impairment of bone microstructure and upregulation of osteoclastogenic markers in spontaneously hypertensive rats

**DOI:** 10.1038/s41598-019-48797-8

**Published:** 2019-08-23

**Authors:** Wacharaporn Tiyasatkulkovit, Worachet Promruk, Catleya Rojviriya, Phakkhananan Pakawanit, Khuanjit Chaimongkolnukul, Kanchana Kengkoom, Jarinthorn Teerapornpuntakit, Nattapon Panupinthu, Narattaphol Charoenphandhu

**Affiliations:** 1 Department of Biology, Faculty of Science, Chulalongkorn University, Bangkok, 10330 Thailand; 2 Center of Calcium and Bone Research (COCAB), Faculty of Science, Mahidol University, Bangkok, 10400 Thailand; 3 Department of Physiology, Faculty of Science, Mahidol University, Bangkok, 10400 Thailand; 4 Synchrotron Light Research Institute (Public Organization), Nakhon Ratchasima, 30000 Thailand; 5 National Laboratory Animal Center, Mahidol University, Nakhon Pathom, 73170 Thailand; 6 Department of Physiology, Faculty of Medical Science, Naresuan University, Phitsanulok, 65000 Thailand; 7 Institute of Molecular Biosciences, Mahidol University, Nakhon Pathom, 73170 Thailand; 8 The Academy of Science, The Royal Society of Thailand, Dusit, Bangkok, 10300 Thailand

**Keywords:** Bone, Calcium and phosphate metabolic disorders

## Abstract

Hypertension and osteoporosis are the major non-communicable diseases in the elderly worldwide. Although clinical studies reported that hypertensive patients experienced significant bone loss and likelihood of fracture, the causal relationship between hypertension and osteoporosis has been elusive due to other confounding factors associated with these diseases. In this study, spontaneously hypertensive rats (SHR) were used to address this relationship and further explored the biophysical properties and the underlying mechanisms. Long bones of the hind limbs from 18-week-old female SHR were subjected to determination of bone mineral density (BMD) and their mechanical properties. Using synchrotron radiation X-ray tomographic microscopy (SRXTM), femoral heads of SHR displayed marked increase in porosity within trabecular area together with decrease in cortical thickness. The volumetric micro-computed tomography also demonstrated significant decreases in trabecular BMD, cortical thickness and total cross-sectional area of the long bones. These changes also led to susceptibility of the long bones to fracture indicated by marked decreases in yield load, stiffness and maximum load using three-point bending tests. At the cellular mechanism, an increase in the expression of osteoclastogenic markers with decrease in the expression of alkaline phosphatase was found in primary osteoblast-enriched cultures isolated from long bones of these SHR suggesting an imbalance in bone remodeling. Taken together, defective bone mass and strength in hypertensive rats were likely due to excessive bone resorption. Development of novel therapeutic interventions that concomitantly target hypertension and osteoporosis should be helpful in reduction of unwanted outcomes, such as bone fractures, in elderly patients.

## Introduction

Hypertension and osteoporosis are major public health problems affecting aging population worldwide. These two chronic diseases account for significant morbidity and mortality, which are related to complications of the diseases, e.g., stroke and fracture, respectively^1–3^. Genetics, aging, lifestyle and environmental factors contribute to the pathogenesis of the diseases, in which many factors are shared^4–6^. Interestingly, clinical studies in hypertensive patients have demonstrated that high blood pressure was associated with low bone mineral density (BMD) with increasing risk of fracture in these patients^7–9^. These evidence highlight that high blood pressure and bone loss are not two independent outcomes in a single patient. However, further investigation is required to establish bone loss and osteopenia as parts of many unwanted consequences of hypertension.

Since the etiologies of these two diseases are closely related and often co-exist with certain factors, such as aging, improper nutritional status, unhealthy lifestyle, lack of exercise, and physical inactivity^6,10^. These factors can individually deteriorate both cardiovascular and skeletal functions. Thus, it is critical to independently assess the relationship of these two diseases without other confounding factors. Animal model of hypertension should provide good platforms for testing this relationship because the cause of hypertension should be well-defined without any influence from other confounding factors. Spontaneously hypertensive rats (SHR)—a hypertensive animal model developed by selective breeding Wistar-Kyoto individuals carrying sporadic gene mutations linked to blood pressure control^11^—were used in the present study. This SHR model has been widely used as a model of essential hypertension with the onset of high blood pressure at 4–6 weeks and cardiovascular complications at 24 weeks of age^12^. Therefore, these rats should allow us to address any changes in the skeleton as outcomes of genetic-induced high blood pressure with controlled environmental variables.

However, there are conflicting reports on skeletal changes in the hypertensive animal models. For example, loss of trabecular thickness and impaired bone healing at proximal tibia were observed in 12-week-old male SHR^13^. In addition, male SHR displayed trabecular loss and delayed healing at the dental socket of mandibles^14^. In contrast, improved trabecular BMD and microarchitecture were reported in 20-month-old male SHR^15^. One of the objectives in this study is to address the discordant outcomes using comprehensive approaches for examining bone mass, density, mechanical properties in SHR compared to age/sex-matched normotensive controls.

Bone is a dynamic tissue and constantly being remodeled to maintain optimal mass and integrity throughout life, in which bone resorption and formation are kept in balance. Bone forming cells, osteoblasts, are differentiated from mesenchymal stem cells under the control of transcription factors, *runt*-related transcription factor (Runx) 2 and osterix^16^. Later, it produces collagen and other proteins including alkaline phosphatase (ALP) and osteocalcin, that are necessary for bone matrix synthesis and mineralization. In addition, these cells also secrete soluble factors, such as receptor of nuclear factor- κB ligand (RANKL), osteoprotegerin (OPG), macrophage colony stimulating factor (M-CSF) and interleukin (IL)-6, to direct differentiation of bone-resorbing cells, osteoclasts^17,18^. Whether these factors produced by osteoblasts are altered due to high blood pressure requires further investigation. Overall, complete characterization of these skeletal changes with underlying cellular mechanisms would be benefit for establishing the use of this *in vivo* pre-clinical model for development of dual therapy targeting hypertension and osteoporosis.

## Materials and Methods

### Animals

Seventeen-week-old of female spontaneously hypertensive rats (SHR/KyoMlac or SHR) and age/sex-matched normotensive wild-type rats (WMN/NrsMlac or WT) were obtain from the National Laboratory Animal Centre of Thailand (NLAC), Mahidol University. Upon arrival at the Central Animal Facility, Faculty of Science, Mahidol University (MUSC-CAF), animals were acclimatized for 5 days and general health status was checked daily by a veterinarian. At the age of 18 weeks old, blood pressure of all animals was monitored using non-invasive tail-cuff method (CODA^®^ tail-cuff blood pressure system, Kent Scientific Corporation, USA). All rats were divided into two groups, i.e., hypertensive and normotensive, based on systolic and diastolic pressure. Animals were housed under 12 h/12 h light-dark cycle during the experimental period. They were fed with standard diet containing 1.0% calcium, 0.9% phosphorus and 4000 IU/kg vitamin D (CP Co., Ltd., Thailand) and reverse osmosis water ad libitum. Room temperature and humidity was controlled at 21–23 °C and 30–70%, respectively. The experimental protocol was approved by the Institutional Animal Care and Use Committee (IACUC), Faculty of Science, Mahidol University and all experiments were performed in accordance with relevant guidelines and regulations.

### Synchrotron radiation X-ray tomographic microscopy (SRXTM) of femoral heads

Fresh frozen femoral heads from SHR and WT controls were collected and used in the present study. Each sample was placed in a cylindrical sample holder filled with cottons soaked in formaldehyde solution to prevent displacement and dehydration during tomographic scanning. SRXTM experiments were performed at the XTM beamline (BL1.2 W: X-ray imaging and tomographic microscopy), Synchrotron Light Research Institute (SLRI), Nakhon-Ratchasima, Thailand. The synchrotron radiation was generated from 2.2-Tesla multipole wiggler in the Siam Photon Source operated at 1.2 GeV at 150 mA. The SRXTM imaging of femoral heads were carried out using filtered polychromatic X-ray beam with a maximum area of 4 × 8 mm^2^ at 10 keV, at a distance of 34 m from the source. The sample projections were obtained from the detection system, which was equipped with 200-µm-thick YAG:Ce scintillator, the white-beam microscope (Optique Peter, France) and the pco.edge 5.5 sCMOS camera (2560 × 2160 pixels, 16 bits). All tomographic scans were acquired at a pixel size of 1.44 µm, which provides a field of view of 3.1 × 3.70 mm^2^. In order to resolve fine details of femoral head, a tomographic volume was reconstructed from enlarged composite projections acquired from two scans. The first scan was taken over 180°. Then, the other 180° scan was taken on vertical axis of rotation that shifted horizontally and parallel to the camera^19^. Subsequently, the X-ray projections were normalized by flat-field correction, stitched, and reconstructed using Octopus software as described previously^20^. All 3D representation of tomographic volume (typically 2000 slices) was rendered using Drishti software^21^.

### Measurement of bone microarchitecture and density

Micro-computed tomography (µCT; model 1178, SkyScan, Aartselaar, Belgium) was used to examine trabecular and cortical bone volume as previously described^22^. The equipment was equipped with X-ray tube voltage of 65 kV, current of 615 lA with 0.5-mm aluminum filter. The scanning angular rotation was 180° with angular increment in 0.54°. The volume of interest (VOI) was between 1.43 and 1.86 mm spanning the proximal and distal growth plates of long bones (50 slides). Images were reconstructed and analyzed by a computer cluster running SkyScan CT-analyzer software package (version 1.11.10).

### Measurement of osteoclast number and active erosion surface

The OsteoMeasure histomorphometric system (version 4.1; Osteometric Inc., Atlanta, GA) with a light microscope (model BX51TRF; Olympus, Tokyo, Japan) was used to visualize osteoclast morphology, and determine number of osteoclasts normalized by tissue area (Oc.N/T.Ar, mm^−2^) as well as active erosion surface normalized by bone surface (aES/BS, %), the latter of which is a proxy indicator of osteoclast activity. The tibiae from WT and SHR groups were cleaned of adhering tissues and were then dehydrated in 70, 95 and 100% vol/vol ethanol for 3, 3, and 2 days, respectively, as described previously^23^. Dehydrated bone specimens were then embedded in methyl methacrylate resin at 42 °C for 48 h. The resin-embedded specimens were first adjusted to obtain the same orientation. Then, they were cut longitudinally to obtain 7-mm thick sections by using a rotary microtome equipped with a tungsten carbide blade (model RM2255; Leica, Nussloch, Germany). Thereafter, each section was mounted on a standard microscope slide, deplastinated, dehydrated, and processed for Goldner’s trichrome staining^23^. The region of interest (ROI) covered the entire trabecular area of the proximal tibial metaphysis at 1–2 mm distal to the growth plate (i.e., secondary spongiosa).

### Assessment of bone mechanical properties

Three-point bending technique was used to evaluate the flexional stiffness and strength of femur (model 5943, Instron, Norwood, MA, USA). The femoral length and thickness at mid-shaft were recorded before each mechanical test. Cross-head displacement rate was 2 mm/min. Test was conducted on mid-diaphysis of femur, which was placed in two supports 20 mm apart and with the femoral anterior margins facing down-ward toward the actuator. Load-displacement curves of each specimen were constructed by Instron5900 software (Norwood, MA, USA). The recorded parameters were ultimate load, yield load and stiffness, which could represent bone strength.

### Osteoblast isolation and primary osteoblast culture

Right tibiae and fibulas were collected and extraneous soft connective tissue was removed from the outer surface using a scalpel blade. Intact bones were rinsed with phosphate-buffered saline (PBS, pH 7.4) and transferred to a petri dish containing Dulbecco’s modified Eagle’s medium (DMEM, Sigma) with 100 IU/ml penicillin and 100 μg/ml streptomycin (Gibco) under sterile condition. The bone sample was then cut into small fragments of 1–3 mm^2^ and washed in 10 ml of DMEM until bone marrow contents were completely clear. Bone fragments were transferred to T75 culture flask containing 30 ml DMEM with 0.25% collagenase (Sigma) and incubated at 37 °C for 4 h. To stop the digestion, DMEM containing collagenase was replaced by DMEM with 10% fetal bovine serum (FBS; Sigma). The solution containing bone chips was then transferred to a new T75 culture flask with growth medium containing 30 ml DMEM supplemented with 30% FBS, 100 IU/ml penicillin and 100 μg/ml streptomycin, 50 μg/ml l-ascorbate-2-phosphate and 100 μM sodium pyruvate (Gibco). Bone cell cultures were incubated at 37 °C in 5% CO_2_ for 7 days with DMEM supplemented with growth medium containing 15% FBS. Growth medium was changed at day 3 and 6 prior to further experiments.

### RNA isolation and quantitative real-time RT-PCR

Total RNA was isolated from primary osteoblasts using TRIZol reagent (Invitrogen, Carlsbad, CA, USA), according to the manufacturer’s instruction. Purity of the total RNA was evaluated by NanoDrop-2000c spectrophotometer (Thermo Scientific, Waltham, MA, USA) reading at 260 and 280 nm, the ratio of which ranged between 1.8 and 2.0. Then, 1-µg total RNA was reverse-transcribed to cDNA with iScript cDNA synthesis kit (Bio-rad, Hercules, CA, USA) using conventional thermal cycler (modelMyCycler, Bio-rad). β-actin was used as an internal control to check the consistency of reverse transcription (percent coefficient of variation < 5%; n = 6–8). Rat PCR primers used in this study are shown in Table 1. The primers have been validated for specificity and efficiency by conventional RT-PCR, as previously described^24,25^. Conventional PCR was performed with GoTaq Green Master Mix (Promega, Madison, WI, USA). PCR products were visualized on 2% agarose gel stained with 1 mg/ml ethidium bromide (Sigma) under a UV transilluminator (Alpha Innotech, San Leandro, USA). The qRT-PCR and melting curve analyses were performed by QuantStudio 3 real-time PCR system (Applied Biosystems, Foster City, CA, USA) with SsoFast EvaGreen Supermix (Bio-rad) for 40 cycles at 95 °C for 60 s, 51–60 °C annealing temperature for 30 s, and 72 °C for 30 s. Relative expression were calculated from the threshold cycles (C_t_) based on the standard ΔC_t_ method.

**Table 1:** *Rattus norvegicus* oligonucleotide sequences used in PCR experiment.

Gene	Accession no.	Primer (Forward/Reverse)	Product size (bp)	Annealing temperature (°C)
*Osteoblast differentiation markers*				
Runx2	NM_053470	5′–TAACGGTCTTCACAAATCCTC–3′ 5′–GGCGGTCAGAGAACAAACTA–3′	135	54
Osterix	AY177399	5′–GCCTACTTACCCGTCTGA–3′ 5′–CTCCAGTTGCCCACTATT–3′	139	55
ALP	NM_013059	5′–AGAACTACATCCCCCACG–3′ 5′–CAGGCACAGTGGTCAAGGT–3′	144	58
Osteocalcin	J04500	5′–CACAGGGAGGTGTGTGAG–3′ 5′–TGTGCCGTCCATACTTTC–3′	203	57
Collagen type I	NM_053304.1	5′–CAGTCGATTCACCTACAGCAC–3′ 5′–GGGATGGAGGGAGTTTACACG–3′	194	59
*Osteoblast*-*derived osteoclastogenic factors*				
M-CSF	NM_023981	5′–ATCCAGGCAGAGACTGACAGA–3′ 5′–CGCAGTGTAGATGAACCATCC–3′	182	55
IL-6	NM_012589	5′–GCAAGAGACTTCCAGCCAGT–3′ 5′–AGCCTCCGACTTGTGAAGTG–3′	145	54
RANKL	NM_057149	5′–TCGCTCTGTTCCTGTACT–3′ 5′–AGTGCTTCTGTGTCTTCG–3′	145	53
*Anti*-*osteoclastogenic factor*				
OPG	NM_012870	5′–ATTGGCTGAGTGTTCTGGT–3′ 5′–CTGGTCTCTGTTTTGATGC–3′	140	53
*Housekeeping gene*				
β-actin	NM_031144	5′–CAGAGCAAGAGAGGCATCCT–3′ 5′–GTCATCTTTTCACGGTTGGC–3′	185	54

Runx2, *runt*-related transcription factor 2; ALP, alkaline phosphatase; M-CSF, macrophage colony-stimulating factor; IL, interleukin; RANKL, receptor activator of nuclear factor-κB ligand; OPG, osteoprotegerin.

### Statistical analysis

The results are expressed as means ± standard error of mean (SEM). The data were analyzed by unpaired Student’s *t*-test. The statistical significance was considered when *P* < 0.05. All data were analyzed by GraphPad Prism 5 (GraphPad, San Diego, CA, USA).

## Results

### SHR exhibited significant elevation of blood pressure

Longitudinal assessment of blood pressure parameters in SHR revealed progressive elevation of systolic and diastolic pressure reaching statistical significance at five to six weeks old in both sexes. In this study, we assessed systolic and diastolic pressure of our SHR rats at 18 weeks old and also found that both parameters were significantly elevated (Table 2). The ranges of systolic and diastolic pressure were consistent with the previous report^11,26,27^.

**Table 2:** Systolic and diastolic blood pressure of SHR and WT (normotensive rats).

	n	Systolic pressure (mmHg)	Diastolic pressure (mmHg)
WT	8	117.94 ± 9.61	74.29 ± 8.24
SHR	8	156.81 ± 9.42**	107.93 ± 9.69**

WT, wild-type; SHR, spontaneously hypertensive rats.

**P < 0.01 compared with WT.

### SHR displayed low bone mass and density

**Figure 1:**
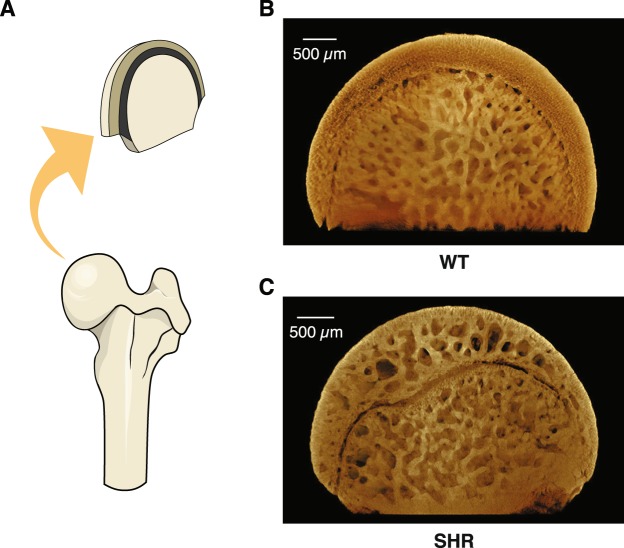
The Synchrotron radiation X-ray tomographic microscopy (SRXTM) imaging of femoral heads of femur. The cross-sectional structure of femoral head (**A**), the microstructural changes of femoral head in 18-week WT controls (**B**) and SHR (**C**).

Having confirmed the hypertensive phenotypes in these SHR, we first performed a high-resolution screening for the changes on bone parameters associated with elevated blood pressures using SRXTM. This technique allowed us to visualize changes in bone structure of these rats in details, superior to those obtained from conventional µCT^28^. Head of femur was chosen as a representative site of fractures as well as hypertension-associated necrosis often observed in the elderly^29^. The 3D structure of femoral heads revealed a marked decrease in bone mass near the epiphysis of SHR. A disarray of the subchondral trabeculae was conspicuous in SHR compared to the WT controls. In addition, the porosity was apparently greater in hypertensive rats than WT rats (Fig. 1).

**Figure 2:**
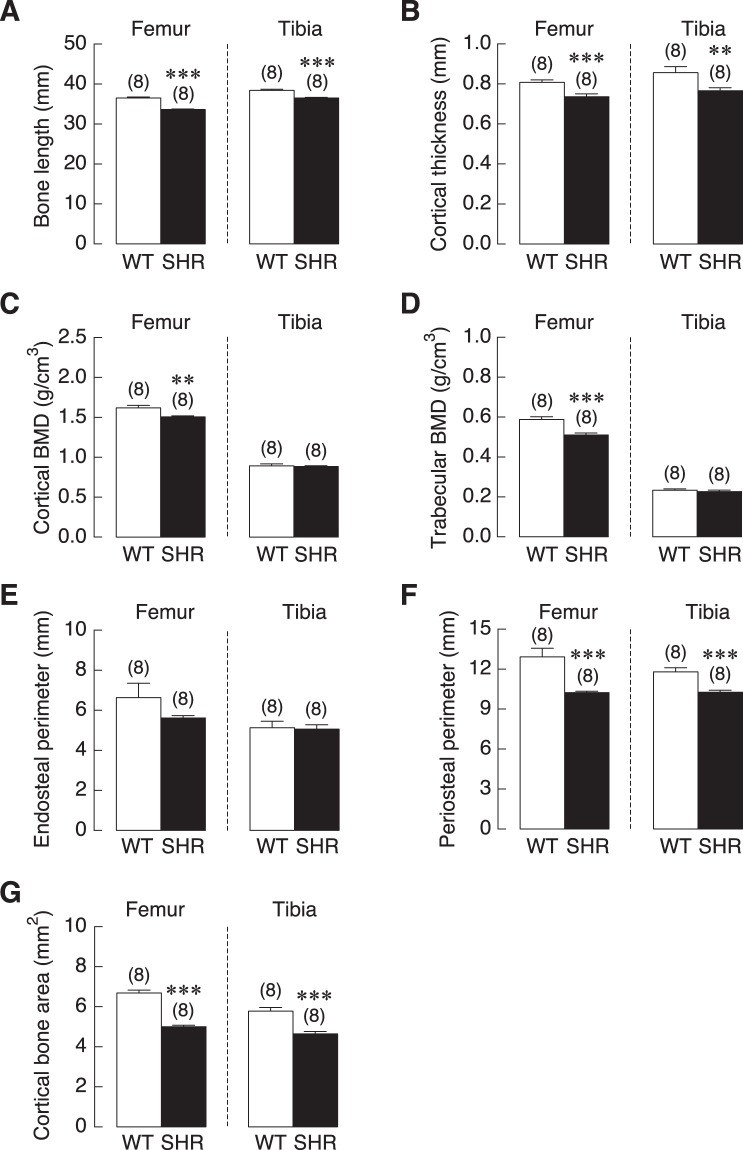
Microstructural parameters of femurs and tibiae of WT controls and SHR. (**A**) Bone length, (**B**) cortical BMD, (**C**) trabecular BMD, (**D**) cortical thickness, (**E**) endosteal perimeter, (**F**) periosteal perimeter, and (**G**) cortical bone area (n = 8 per group). **P < 0.01 and ***P < 0.001 compared with WT.

We then quantified the changes in cortical and trabecular bones using volumetric µCT. Femurs and tibia from 18-week-old female SHR and WT controls were assessed. The total length of each sample was measured and found that both femur and tibia from SHR exhibited a significant decrease in total length (Fig. 2A). For the volumetric measurement of cortical and trabecular bone density, we found that cortical as well as trabecular BMD in the femora of SHR was significantly decreased as compared to WT controls. However, in the tibia, there was no difference in both cortical and trabecular BMD of SHR compared to WT controls (Fig. 2C,D).

We further examined additional parameters of these long bones across cross-sectional planes. Cortical thickness of these two bones was significantly reduced in female SHR compared to WT controls (Fig. 2B). In addition, periosteal perimeter and cortical bone area of femur and tibia from SHR were decreased (Fig. 2F,G), suggesting a decrease in cross-sectional area of the long bones. Taken together, long bones from SHR exhibited decreases in length and width and a reduction in cortical and trabecular BMD compared to the WT controls.

### Reduction in bone mass and density in SHR were associated with a decrease in strength

**Figure 3:**
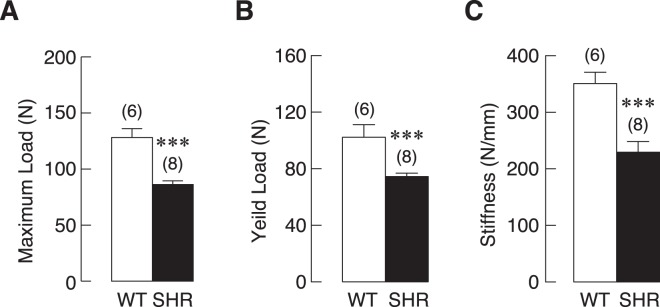
Mechanical property analysis of left femurs of WT controls and SHR. (**A**) maximum load, (**B**) yield load, and (**C**) stiffness (n = 6–8 per group). ***P < 0.001 compared with WT.

We then asked whether those bone defects characterized by µCT analyses compromised the strength in SHR. Three-point bending test was used on femurs from 18-week-old female SHR and WT controls. Long bones from SHR showed a marked reduction in ability to withstand maximum load compared to respective controls (Fig. 3A). Similarly, ability of SHR femurs to withstand the yield load prior to the irreversible deformities was significantly decreased (Fig. 3B). Next, bone stiffness was assessed to further demonstrate the mechanical impairment. Indeed, femurs from SHR exhibited a significant decrease up to 40% compared to those from WT controls. Therefore, our data demonstrated that reduction in bone mass and density in SHR led to impairment of bone strength.

### Changes in expression of osteoblastic genes in SHR

**Figure 4:**
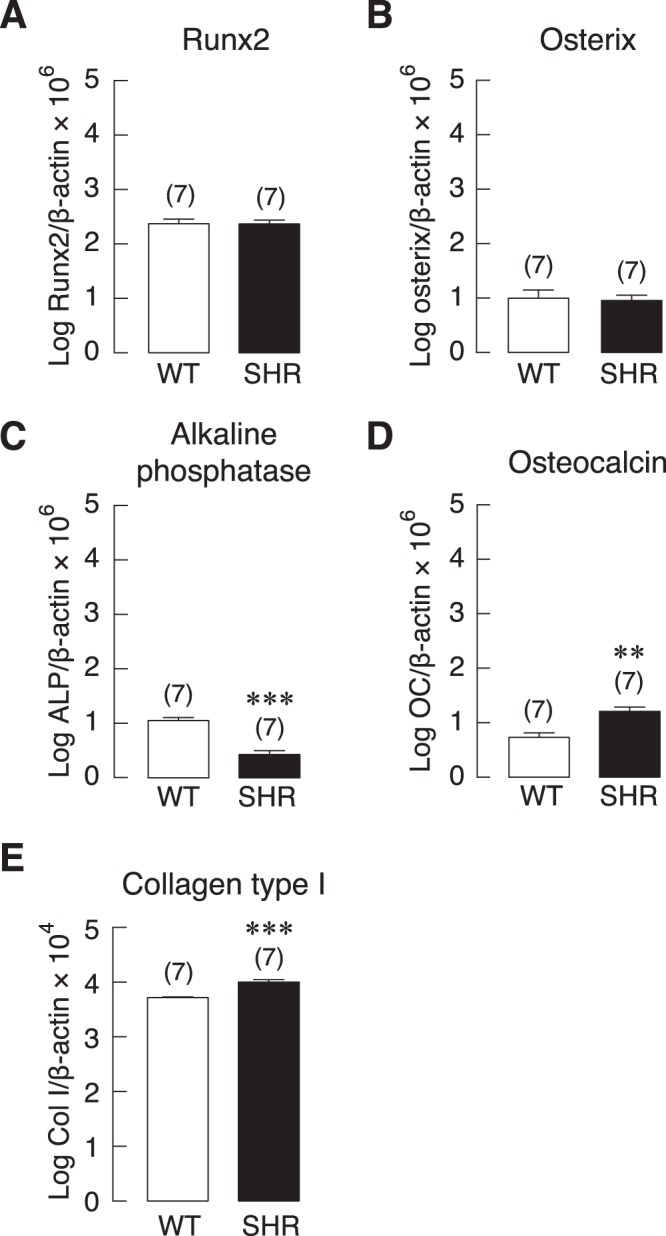
mRNA expression of bone formation markers, i.e., (**A**) Runx2, (**B**) osterix, (**C**) ALP (**D**) osteocalcin, and (**E**) collagen type I (Col I) in primary osteoblasts of WT controls and SHR. The mRNA expression of each gene in seven independent samples (n = 7) was determined by quantitative real-time PCR, and was normalized by β-actin expression. **P < 0.01, ***P < 0.001 compared with WT.

We then investigated the possible mechanisms that linked to decreased bone mass and bone strength in SHR. Primary osteoblasts were isolated from 18-week-old female SHR and WT controls and mRNA expression of markers associated with bone formation and resorption was assessed. First, we examined the expression of genes that promote osteoblast differentiation. There was no significant difference in transcript levels of Runx2 and osterix in osteoblasts from SHR and WT controls (Fig. 4A,B). Next, the expression of genes involving in bone formation was investigated. Expression of ALP was markedly decreased in the cells from SHR compared to WT controls (Fig. 4C). Osteoblasts from SHR expressed slightly higher but significant levels of osteocalcin and collagen type I transcripts compared to those from WT controls (Fig. 4D,E). This discrepancy in regulation of bone formation and mineralization suggested that other regulatory markers of bone remodeling play critical roles in the bone phenotypes of SHR.

**Figure 5:**
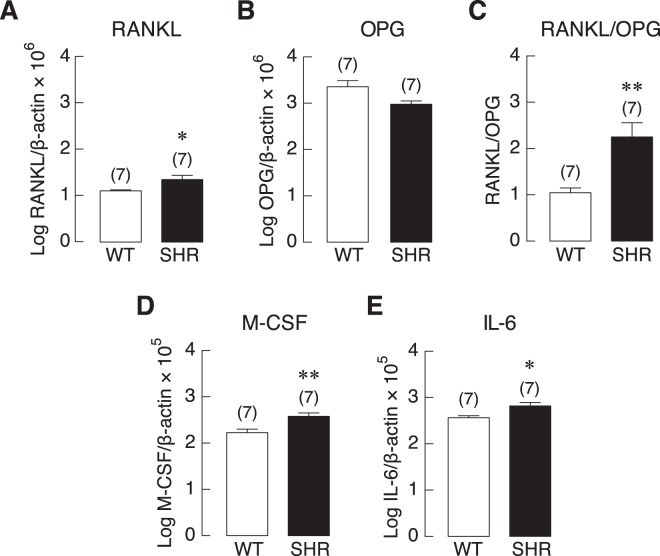
The mRNA expression levels of osteoblast-derived osteoclastogenic factors in primary osteoblasts of SHR and normotensive rats (WT). (**A**) RANKL, (**B**) OPG, (**C**) RANKL/OPG ratio, (**D**) M-CSF, and (**E**) IL-6. The mRNA expression of each gene in seven independent samples (n = 7) was determined by quantitative real-time PCR, and was normalized by β-actin expression. *P < 0.05, **P < 0.01 compared with WT.

Since osteoblasts are able to regulate osteoclast differentiation and activity via soluble factors and cytokines, we then examined the expression of these osteoblastic genes. We found that expression of RANKL was significantly upregulated in SHR (Fig. 5A). Expression of OPG was also reduced without reaching statistical significance in these rats (Fig. 5B). Together, the ratio of RANKL/OPG expression was markedly increased in SHR compared to WT controls (Fig. 5C). Furthermore, expression of other osteoclastogenic factors, M-CSF and IL-6, was significantly increased in SHR (Fig. 5D,E, respectively). Together, these data suggested that dysregulation of bone remodeling process, i.e., reduced bone formation and increased bone resorption, led to the reduction in bone mass and strength of those hypertensive rats.

### Active erosion surface was increased in SHR without change in osteoclast number

**Figure 6:**
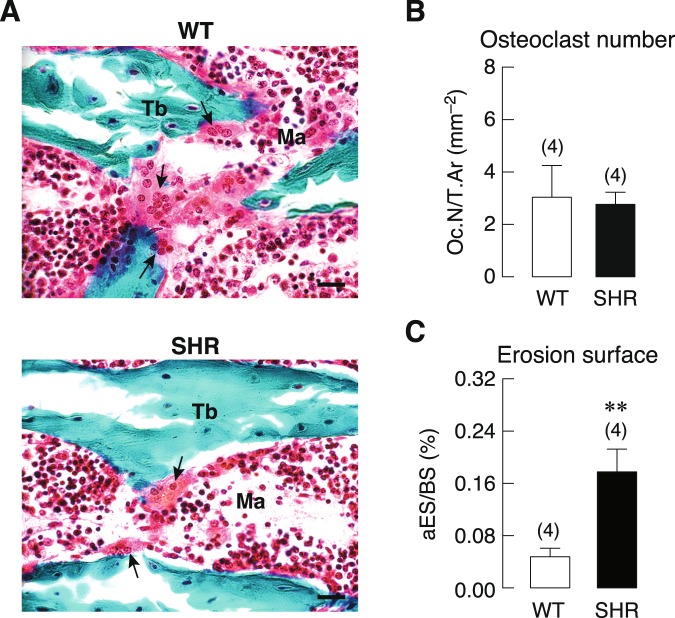
(**A**) Representative photomicrographs of large multinucleated cells showing normal morphology of osteoclasts (*arrows*) in SHR and WT rats (bars = 20 μm). Tb, bone trabeculae; Ma, marrow cavity. (**B**) Osteoclast number normalized by tissue area (Oc.N/T.Ar) and (**C**) active erosion surface normalized by bone area (aES/BS) of proximal tibial metaphyses in SHR and WT rats (n = 4), as determined by computer-assisted OsteoMeasure histomorphometric system. Numbers in parentheses are numbers of animals per group. **P < 0.01 compared with WT.

Since the upregulation of RANKL, M-CSF and IL-6 expression (Fig. 5) suggested that there was an increase in osteoclastogenesis and/or osteoclast activity, we determine the osteoclast number (Oc.N/T.Ar) and active erosion surface (aES/BS), which are proxy indicators of osteoclast precursor cell proliferation and osteoclastic activity, respectively, in SHR and WT rats. As shown in Fig. 6A, differentiated osteoclasts from both SHR and WT groups had the same morphology (i.e., large multinucleated cells residing on the trabecular surface). However, the number of osteoclasts of SHR was not different from that of normotensive WT rat (Fig. 6B). Interestingly, the active erosion surface was significantly enlarged in SHR (Fig. 6C), suggesting that hypertension was associated with the enhanced osteoclast differentiation and/or activity rather than proliferation of pre-osteoclast into multinucleated cell.

## Discussion

In this study, we have demonstrated that high blood pressure in SHR caused marked reduction of bone mineral density at both cortical and trabecular sites as decrease in cross-sectional area of the long bones. Importantly, these changes led to substantial decreases in strength to withstand external forces as indicated by three-point bending tests. Further, we found that these rats manifested the increased bone resorption markers concomitantly with decreased bone formation makers. These data have suggested that high blood pressure led to compromised bone structure and mechanics, which might, in turn, result in a greater incidence of fracture.

Previous studies were often carried out using male SHR particularly when characterizing bone phenotypes. Less is known about bone phenotypes in female SHR although the incidence of bone loss and fracture are in fact greater in women. Interestingly, strong correlation between hypertension and bone loss was found selectively in female subjects with a significant increase in overall cumulative incidence of fracture^30^. To examine early changes in the skeleton due to hypertension, we thus used young adult female rats at the age of 18 weeks. These rats fully developed hypertension without reproductive senescence^31^; therefore, we were able to avoid the interference owing to age-dependent changes in gonadal steroids. Here, our data indicated that female SHR were susceptible to fracture due to compromised bone mass, mimicking those found later on in aging population, particularly in the postmenopausal group. Consistent with our study, ovariectomized adult female SHR displayed accelerating loss of cortical and trabecular bones compared to controls^32^. Further, it would be interesting to examine mechanical properties of bone in these ovariectomized and aged SHR.

Due to the limitations of the imaging resolution in µCT technique, it has still not been possible to fully characterize bone micro-architecture, i.e., porosity. Therefore, in this study, synchrotron imaging techniques have been developed to overcome this limitation in spatial resolution^28^. We utilized two techniques, SRXTM and conventional µCT to comprehensively examine long bones of the hind limb, particularly at the changes in bone mass, density and microarchitecture. High-resolution images from SRXTM indicated an increase in porosity of the femoral head from SHR, which was similar to those observed in osteoporotic patients^33,34^. This decrease in trabecular bone connectivity is likely to associate with tendency to fracture especially at the femoral neck, a common fracture site with long-term health consequences. Indeed, hypertensive patients had higher risk of the femoral neck fracture compared to their cohorts^6^. Furthermore, avascular necrosis of the femoral head is another common complication associated with hypertension, thereby aggravating poor bone quality. Specifically, it has been shown that stroke-prone SHR with systolic pressure of 250 mmHg and end organ damages developed avascular necrosis at the head of femur at the age of 13 weeks^35^. Nevertheless, our SRXTM and µCT images from SHR at the age of 18 weeks without extremely high systolic pressure showed intact femoral heads without irregular contour, a sign of avascular necrosis. However, it is still possible that long-term moderate hypertension seen in our SHR model might eventually lead to progressive necrosis of the femoral head. Collectively, these findings point to increases in porosity and fracture tendency owing to poor bone quality in the long bones from this SHR model.

Our study has demonstrated for the first time that long-standing hypertension resulted in marked reduction in resistance to external forces. Here, we found that ability of bone to withstand loads was dramatically decreased in SHR group reflecting a decrease in bone strength and increase tendency to fracture. Bone strength is determined by the degree of mineralization, cortical and trabecular architecture, and rate of bone remodeling^36^. Aberrant regulation of bone quality—i.e., degree of bone mineralization as well as synthesis and alignment of bone matrix—may compromise the strength^37^. Therefore, bone fragility in SHR model could probably link to mineralization defects or misconfiguration of collagen alignment. It would be interesting to further examine whether these structural defects exist in SHR skeleton. Defects of collagen alignment and other defects related to connective tissue microstructures in bone and blood vessel may underlie the common etiologies of bone fragility and cardiovascular dysfunction found in SHR^38^.

Maintenance of the bone mass reflects the balance of osteoblast-mediated bone formation and osteoclast-mediated resorption. The exact underlying mechanism by which hypertension induces bone loss remains unclear. However, a number of factors were identified in hypertensive condition—such as overproduction of proinflammatory cytokines and reactive oxygen species^39,40^, which could enhance osteoclast functions and/or suppress osteoblast function. In this regard, we hypothesized that high blood pressure compromised the integrity of bone tissue via dysregulation of bone cell functions at the cellular and molecular levels. At early osteoblast development from mesenchymal cells to osteoblastic lineage, Runx2 and osterix are salient transcription factors for both osteoblast proliferation and differentiation under normal conditions and perhaps hypertensive condition as well. Once osteoblast proliferation declines, the expression of an early marker of matrix maturation phase, particularly ALP, is gradually increased. Finally, bone matrix is mineralized concurrently with an increase in the expression level of osteocalcin, which is the major non-collagenous protein in bone matrix essential for calcium biding, stabilization of hydroxyapatite in the matrix, and regulation of bone formation^41^. Since our results showed an absence of changes in Runx2 and osterix expression in hypertensive rats whereas a decrease in ALP expression together with increases in osteocalcin and collagen type I expression were found, it was suggested that the pre-osteoblasts from SHR group had similar potential to proliferate and start to differentiate into mature osteoblasts. Nevertheless, when the process of differentiation commenced, the SHR osteoblasts might have less ability to fully differentiate or slowdown in the process of differentiation, as suggested by downregulation of ALP, a marker of early differentiated cells. Thereafter, once the osteoblasts fully differentiated, they might resume their function and were thus able to express osteocalcin and collagen type I, the upregulation of which in SHR probably a sign of compensation to help overcome hypertensive osteopathy.

It is also possible that some other hypertension-induced impairments of osteoblastic functions, e.g., a downregulated ALP expression—which eventually impaired mineralization—and malalignment of collagenous matrix might predominate over the compensatory responses, leading to deterioration of bone mechanical properties^42,43^. Moreover, despite being an important osteoblast differentiation marker, it is difficult to predict the long-term consequence of osteocalcin overexpression. Indeed, the expression of osteocalcin has been known to be more variable dependent on various factors, including prolonged exposure to catecholamines and high blood glucose^44,45^. For example, epinephrine and norepinephrine have been reported to upregulate the osteocalcin expression in osteoblasts of male ICR mice^44^. Further investigation is thus required to evaluate the outcome of osteocalcin production from osteoblasts in SHR.

Furthermore, our results showed that high blood pressure led to increases in the expression of RANKL/OPG ratio, IL-6 and M-CSF, all of which could promote osteoclast formation and activity that positively influencing bone resorption. Normally, mature osteoclasts are multinucleated cells of hematopoietic origin with a unique in their ability to resorb bone matrix. Osteoclastogenesis is principally stimulated by two essential cytokines expressed by osteoblasts, i.e., M-CSF and RANKL. The biological activity of RANKL is counterbalanced by the osteoblast-derived decoy receptor, osteoprotegerin (OPG). The expression of RANKL and OPG must be coupled and kept in balance to ensure an equal bone resorption and formation Hence, an increase in RANKL/OPG ratio, as in the present study (Fig. 5), was postulated to enhance osteoclast differentiation and bone resorption^46^. In addition, pro-inflammatory cytokines, especially interleukin-6 (IL-6), are potent inducers of osteoclast activity^47^. Increased circulating levels of M-CSF and IL-6 during hypertension have been reported in both humans and rodents^48,49^, consistent with the present PCR study. In addition, an increase in active erosion surface in SHR without change in osteoclast number suggested that hypertension accelerated the activity of differentiated osteoclasts to resorb bone rather than inducing pre-osteoclast proliferation.

Regarding the osteoblast-mediated bone formation, hypertension might predominantly affect ALP expression, thereby posing a difficulty for osteoblasts to obtain inorganic phosphate for matrix mineralization. This postulation is supported by the fact that ALP is responsible to cleave the phosphate group from phosphorylated proteins in order to supply inorganic phosphate for hydroxyapatite formation. The collagen fibril malalignment as reported previously probably contributes to mineralization defect despite having a sign of collagen type I overexpression^43^. It is also possible that changes in osteoblastic gene expression were cell-autonomous without the presence of high blood pressure. Specifically, genetic predisposition of hypertension might be sufficient to induce osteogenic defect since reduction of osteogenic markers was observed in differentiated bone marrow mesenchymal stem cells from 4-week-old prehypertensive SHR^50^. Further studies are still needed to explore whether the changes in the expression profile were predisposed by genetic alterations and/or the changes in bone microenvironment affected by high blood pressure.

In conclusions, we have demonstrated the effects of high blood pressure on the skeleton of adult female rats using integrative biophysical and molecular approaches. These hypertensive rats have low bone density, presumably due to dysregulation of bone remodeling together with compromised bone architecture susceptible to fracture. Establishment of this hypertensive-induced osteoporosis and fracture model would be valuable for development of new therapeutic regimens for dual treatment of high blood pressure and hypertension-associated osteoporosis.
